# Supplementary material for: Application of naïve Bayesian approach in detecting reproducible fatal collision locations on freeway
Source: PLoS One. 2021 May 18;16(5):e0251866. doi: 10.1371/journal.pone.0251866 (PMC8130948; doi:10.1371/journal.pone.0251866)
Supplement: S1 Dataset — (ZIP) [file pone.0251866.s001.zip › Description of sample data set.docx]

**1. Data availability**

The data set to replicate this study are uploaded as Supporting Information.

**2. Description**

This document provides the description of the traffic collision data collected between 2004 and 2008 from 390 miles of six freeway routes in the San Francisco bay area, including I-80W, I-80E, I-580W, I-580E, I-880N, and I-880S. In California, all vehicle collisions occurred on a public road-way are reported into State wide integrated traffic records system (SWITRS), which is owned and maintained by the California Highway Patrol (CHP). The information about the collisions occurred on Caltrans-owned facilities is then sent to Traffic Accident Surveillance and Analysis System (TASAS), and this has been employed as the collision data source in this study. The provided data set are classified by six routes and 5 years, and the postmile in the data is absolute postmile provided by PeMS (http://pems.dot.ca.gov/)

| Column number | Column name | Description |
| --- | --- | --- |
| 1 | str_pm | Starting postmile of the sites |
| 2 | end_pm | Ending postmile of the sites |
| 3 | mid_pm | Middle postmile of the sites |
| 4 | fatal | Number of fatal collisions in the site |
| 5 | injury | Number of injury collisions in the site |
| 6 | PDO | Number of PDO collisions in the site |
| 7 | total | Number of collisions in the site |
