# Supplementary material for: Application of naïve Bayesian approach in detecting reproducible fatal collision locations on freeway
Source: PLoS One. 2021 May 18;16(5):e0251866. doi: 10.1371/journal.pone.0251866 (PMC8130948; doi:10.1371/journal.pone.0251866)
Supplement: Attachment — Submitted filename: Response to Reviewers.docx [file pone.0251866.s002.docx]

**PONE-D-21-03606 “Application of Naïve Bayesian Approach in Detecting Reproducible Fatal Collision Locations on Freeway”**

**Authors:** Eui-Jin Kim, Oh Hoon Kwon*, Shin Hyoung Park, Dong-Kyu Kim, and Koohong Chung

The authors really appreciate the anonymous referees who reviewed this paper for their constructive and helpful comments. In this response document, we reponse to each review comments with the label **[Response]**, and the revision in the manuscript is also described with the label **[Manuscript].**

### Journal Requirements:

1. Please ensure that your manuscript meets PLOS ONE's style requirements, including those for file naming.

**[Response]**

We checked the style of the revised manuscripts to meet the PLOS ONE’s requirements.

2. Please add information in both the Methods section and the Data availability statement on how the real dataset was obtained, and whether the authors have permission to share this openly with the publication.

**[Response]**

All the traffic collision data used in this study are based on State Wide Integrated Traffic Records Systems (SWITRS) that are open-source database published by California Highway Patrol (California Highway Patrol, 2013). We added the detail information about how the traffic collision data are collected in the **Site and Data** section, and also stated in the **Data Availability Statement.**

**[Manuscript]**

**(Line 129)** All the traffic collision data used in this study are based on State Wide Integrated Traffic Records Systems (SWITRS) that are open-source database published by California Highway Patrol (California Highway Patrol, 2013). The SWITRS database records all vehicle collisions occurred on a public road-way. Among the traffic collision in SWITRS database, Traffic Accident Surveillance and Analysis System (TASAS) collected the collision data that occurred on a road that is managed by Caltrans, and this study used the TASAS collision data.

**(Data Availability Statement)** All relevant data based on SWITRS database are within the paper and its Supporting Information files.

3. We require you to either (1) present written permission from the copyright holder to publish these figures specifically under the CC BY 4.0 license, or (2) remove the figures from your submission:

**[Response]**

We decide to remove the Google Maps figure from our submission since all the critical informations about each route are provided through the Table 1. Instead, we replace the Fig 1 with the new figure that supports the response to the reviewer.

### Responses to Reviewer 1

1. (1)The research data in this paper are traffic accident data of five similar highways in the United States from 2004 to 2008, and the traffic data are too old to have low reference significance for traffic accident prediction and safety improvement under modern traffic conditions. (2) Also in the article "In 2016 alone, there were 1,389 fatal collisions on 15,181 miles of the state highway systems in California [5]. " This does not affect the content of the article, but it is questionable to use data from the past 10 years to argue and analyze the existing traffic phenomenon.

**[Response]**

(1) Thank you for your comments. The main purpose and contribution of this study is to propose an method for detecting reproducible fatal collision locations, and we used the traffic collision data collected from 2004 to 2008 to verify our methods. The main reason for using past data rather than recent data is to compare the previous methods using SPF and CRP, reported by Kwon et al. (2013), which are carefully calibrated to these past dataset with confirmed hot spots (CHS). Specifically, a high collision concentration location (HCCL) detection methods only can be calibrated or evaluated by the list of CHS (i.e., HCCL confirmed by inspection of transportation engineer after the traffic collisions occurs), and this list are generally not publicly available. Therefore, for a rigorous comparison with the proven previous method, we decided to use the past data though the current trend cannot be reflected. The proposed method can be applied to any routes and years only using the location information of traffic collision data and annual traffic volume, and as shown in Table 5, the proposed method shows the robust performance to 5 years of collision data. Therefore, we may expect generalized performance from the recent data.

**[Manuscript]**

**(line 137)** The purpose of the study is to evaluate the reproducibility of fatal collision locations on freeways based on the collision occurrence data and not depending on the influential factors that are changed over time such as vehicle safety systems, roadway conditions, and traffic conditions. Therefore, to evaluate our method by comparing carefully calibrated previous method, we used these datasets applied in the previous studies [13] rather than recent data.

1. (2) Also in the article "In 2016 alone, there were 1,389 fatal collisions on 15,181 miles of the state highway systems in California [5]. " This does not affect the content of the article, but it is questionable to use data from the past 10 years to argue and analyze the existing traffic phenomenon.

**[Response]**

(2) Thank you for your comments. As you pointed out, the sentence “In 2016 alone, there were 1,389 fatal collisions on 15,181 miles of the state highway systems in California [5]” may be inappropriate to support our background. We removed this sentence and added the figure of annual collision trend in California freeway in the revised manuscripts. Since 2005, the number of collisions has declined until 2013 and then increases again to 2017. In comparison between 2005 and 2017, the number of total collisions was quite higher in 2017, the ratio of injury collisions was slightly higher in 2017, and the ratio of the fatal collision was slightly lower in 2005. This trend indicates that both fatal and injury collisions are still issues to be addressed in spite of advances in vehicle safety systems, roadway design, and various traffic safety-countermeasures.

**[Manuscript]**

**(Line 56)** Fig 1 shows the annual collision trend in California freeway from 2005 to 2017. Since 2005, the number of collisions has declined until 2013 and then increases again to 2017. In comparison between 2005 and 2017, the number of total collisions was quite higher in 2017, the ratio of injury collisions was slightly higher in 2017, and the ratio of the fatal collision was slightly lower in 2005 [4]. This trend indicates that both fatal and injury collisions are still issues to be addressed in spite of advances in vehicle safety systems, roadway design, and various traffic safety-countermeasures [5].

2. The quality of the pictures in this article is vague and the content is difficult to identify.

**[Response]**

Thank you for your comment. In the revised manuscript, we revised some parts of the figures (Fig 1 - Fig. 5) in high resolution, and also added the color or leader lines to improve the visibility and content quality. We rewrited the description of Fig 4 by the revised figures.

**[Manuscript]**

**(Line 297)** When $\alpha$ = 0, the shape of *P*(*F_i_* | *D*) remains horizontal. Higher values of α bring the shape of $P(F_{i}|D)$ to a wide peak near *D* and the value of $P(F_{i}|D)$ drops sharply if the distance from $D$ become more than $L_{c}$, whereas lower values of $\alpha$ bring the shape of $P(F_{i}|D)$ to a narrow peak and the value of $P(F_{i}|D)$ drops gently even if the distance from $D$ is larger than $L_{c}$. The black circles in Fig 4 show the value of $P(F_{i}|D)$ at the postmile = 26 according to $\alpha$, which indicates that the high value of α makes the value of $P(F_{i}|D)$ more sensitive to the distance from $D$.

3. The vehicle technology conditions, road conditions, and traffic conditions of existing traffic activities have developed radically changed, how do the research results of the paper reflect the significance of guidance for reality?

**[Response]**

Thank you for your comment. We agree that your comment is correct. Various vehicle safety systems, roadway conditions, and traffic conditions have changed in the past 10 years. However, the annual trend of total and fatal collisions is still on the rise which indicates that HCCL(or hotspot) still remains in the freeway. The proposed method does not find the HCCL based on the complex human and environmental factors but finds the recurrent fatal collision locations using a probabilistic approach based on the locations and number of collisions. The fact that it does not depend on complex influential factors to collisions is one of the main contributions of this study. Therefore, even if vehicle condition and roadway condition change rapidly, the proposed method still can be used to identify the recurrent fatal collision locations with only the recent three years of data. Nevertheless, a comparison of the proposed method with other recent methods using a recent dataset would be further research subject to verify the generalized performance. In the manuscripts, we discussed these contents on the Concluding remarks sections as a future research subject.

**[Manuscript]**

**(Line 540)** Although advanced vehicle safety technology, advanced highway design, and surrogate safety-measures have developed from the past, the number of total and fatal collisions is still rising, which indicates that the HCCL still remains in the freeway. The proposed method does not find the HCCL based on the complex human and environmental factors but finds the recurrent fatal collision locations using a probabilistic approach based on the locations and number of collisions. In other words, the proposed method can be applied to the recent data since it does not depend on the complex influential factors that change over time. Nevertheless a comparison of the proposed method with other methods using a recent dataset would be further research subject to verify the generalized performance.

### Responses to Reviewer 2

1. In order to address the issue a high false-positive rate to identify the high-collision-concentration locations with fatal collisions in previous studies, the paper developed a method for estimating the proxy measure of the posterior probability and compared the improvements with the SPF-based approach. Their work was detailed and complete.
However, the quality of the pictures in the manuscripts needs to be improved. Resolution of most images are too low.

**[Response]**

Thank you for your comment. In the revised manuscript, we revised some parts of the figures (Fig 1 - Fig. 5) in high resolution, and also added the color or leader lines to improve the visibility and content quality. We rewrited the description of Fig 4 by the revised figures.
